# Fat‐poor leiomyomatous angiomyolipoma arising from renal parenchyma negative for HMB‐45: A case report

**DOI:** 10.1002/ccr3.6771

**Published:** 2022-12-20

**Authors:** Yasuyuki Kobayashi, Shigeki Shimizu, Hiroki Arai, Kyotaro Yoshida, Masahito Honda

**Affiliations:** ^1^ Department of Urology Kinki Central Hospital of Mutual Aid Association of Public School Teachers Itami Japan; ^2^ Department of Diagnostic Pathology Kinki Central Hospital of Mutual Aid Association of Public School Teachers Itami Japan; ^3^ Department of Clinical Laboratory Kinki Central Hospital of Mutual Aid Association of Public School Teachers Itami Japan

**Keywords:** angiomyolipoma, HMB‐45, Melan‐A, renal parenchyma, smooth muscle tumor

## Abstract

Fat‐poor leiomyomatous angiomyolipoma, which is similar to smooth muscle tumors, is positive for smooth muscle markers and melanocytic marker human melanin black 45 (HMB‐45). We report a case of fat‐poor leiomyomatous angiomyolipoma arising from renal parenchyma negative for HMB‐45 diagnosed by combined staining with melanocytic markers HMB‐45 and Melan‐A.

## Graphical abstract

We report a rare case of fat‐poor leiomyomatous angiomyolipoma arising from renal parenchyma negative for human melanin black 45 (HMB‐45) but positive for Melan‐A. Combined staining with HMB‐45 and Melan‐A may be useful in the differential diagnosis of renal tumors composed predominantly of smooth muscle cells.

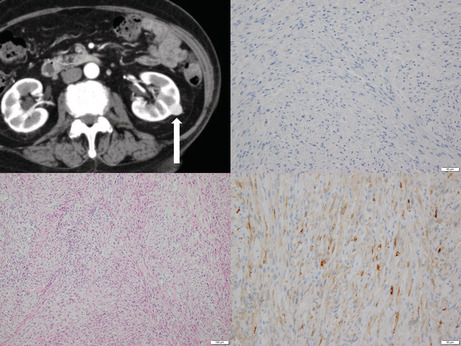

## 1 INTRODUCTION

Angiomyolipoma (AML) is a benign mesenchymal tumor that comprises approximately 1% of all surgically resected renal tumors. AML is composed of a mixture of adipose tissue, smooth muscle, and thick‐walled blood vessels and co‐expresses smooth muscle marker and melanocytic marker.^1^ AML composed of predominantly smooth muscle is confusingly similar to smooth muscle tumors such as leiomyoma, leiomyosarcoma, or gastrointestinal stromal tumor (GIST), and renal cell carcinoma with spindle cell change. Nonomura et al. reported AMLs arising from the renal capsule and liver that were composed almost exclusively of smooth muscle cells and some blood vessels. They designated it leiomyomatous AML and reported HMB‐45 immunostaining to be a useful marker in confirming its diagnosis.^2^ Leiomyomatous AML arising from the renal capsule had no fat cell component and was easily mistaken for leiomyoma.^2^ We have occasionally encountered leiomyomatous AML arising from renal parenchyma (RP),^3^, ^4^, ^5^ but fat‐poor leiomyomatous AML arising from RP is exceedingly rare, with only one previous case reported in English.^6^ We report this rare case of fat‐poor leiomyomatous AML arising from RP that was negative for HMB‐45.

## 2 CASE REPORT

**FIGURE 1:**
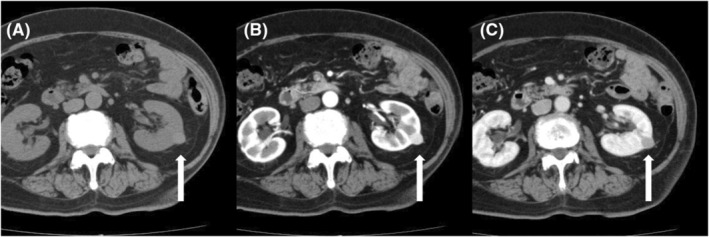
Abdominal computed tomography (CT) images showing the left renal tumor. (A) Unenhanced CT scan shows a homogenous and hyperdense area compared with the normal renal parenchyma. (B) Enhanced CT scan shows homogenous enhancement in the early phase. (C) Enhanced CT scan shows washout in the late phase. White arrows indicate the left renal tumor.

A symptomless left renal mass 15 mm in diameter was found in the lower pole of the left kidney of an 82‐year‐old woman by abdominal computed tomography (CT) screening (Figure 1A). Her history included diabetes mellitus, hypertension, and dyslipidemia but no tuberous sclerosis. Urinalysis was normal, and urinary cytology was negative. Contrast‐enhanced CT showed homogeneous enhancement of the mass with early‐phase contrast effect and late‐phase washout (Figure 1B,C). No metastases were noted. We diagnosed her as having a left renal tumor (cT1aN0M0) and explained partial nephrectomy, ablative techniques, and active surveillance as primary treatments to her. Although she was certainly old, her general condition was good and her Karnofsky performance status was 100%. She wished to undergo curative surgery considering the possibility of cancer. Thus, we performed left laparoscopic partial nephrectomy via a retroperitoneal approach. We identified a plane outside of the tumor and within the normal parenchyma. The renal tumor was nonadherent to the surrounding tissue. We circumferentially incised the renal capsule 5 mm peripheral to the tumor with electrocautery and excised the tumor along with a small rim of normal parenchyma. No apparent residual tumor was observed intraoperatively.

**FIGURE 2:**
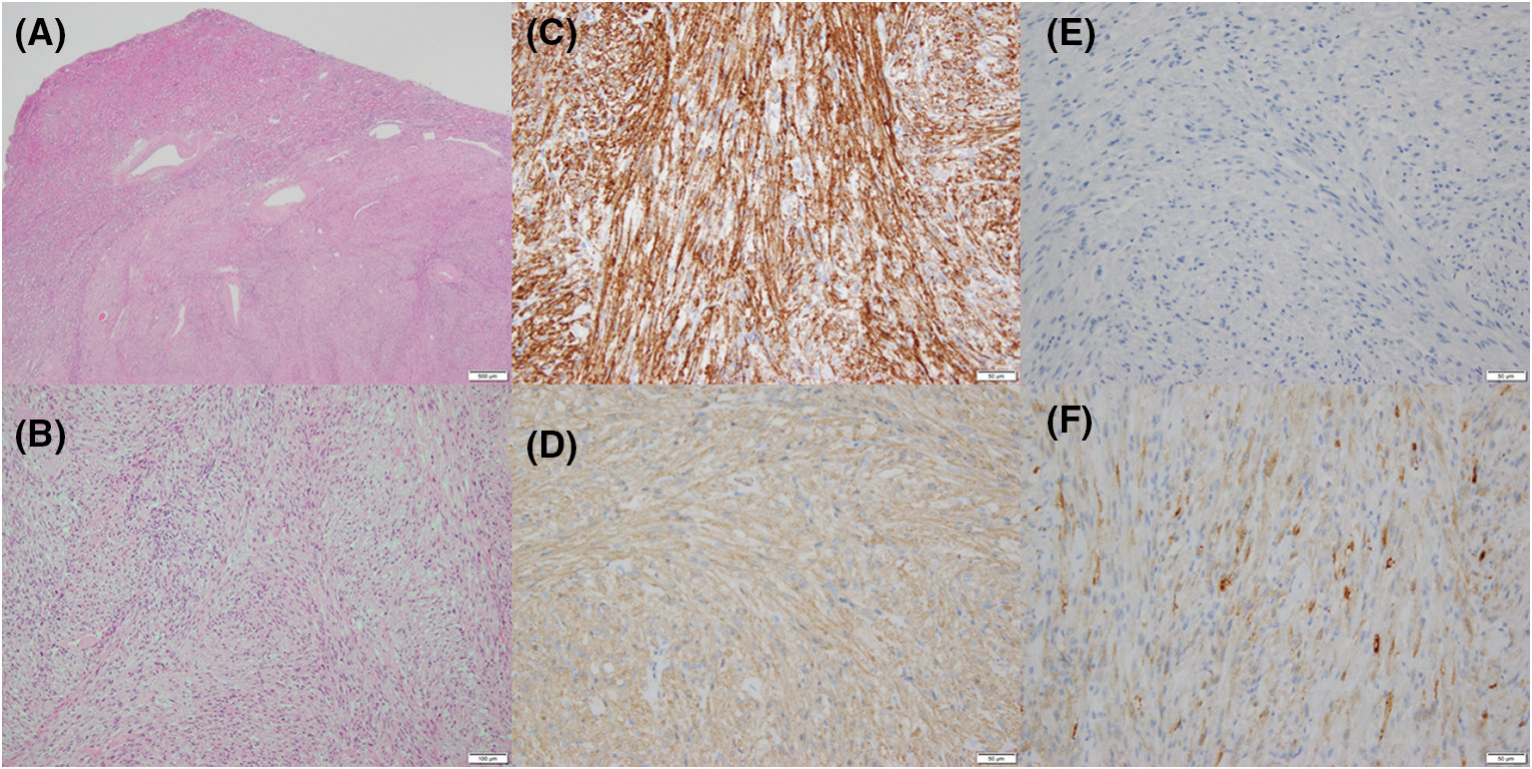
Histopathologic images of the left renal tumor. (A) Hematoxylin and eosin‐stained sections showing the tumor invading the renal parenchyma but not the renal capsule and (B) spindle‐shaped cells and a few vessels without adipocytes. (C) Sections showing Caldesmon‐positive staining, (D) SMA‐positive staining, (E) negative staining for HMB‐45, and (F) Melan‐A‐positive staining.

Macroscopically, the tumor was 15 mm in diameter, yellowish‐white, solid, and poorly circumscribed. Microscopically, it was located in the RP and was nonadherent to the renal capsule. It was composed almost exclusively of spindle‐shaped cells with eosinophilic cytoplasm, ovoid to elongated nuclei with mild atypia and fascicular growth pattern, and contained a few blood vessels and no adipocytes (Figure 2A,B).

Immunohistochemically, the tumor cells were positive for caldesmon, smooth muscle actin (SMA), Melan‐A, S100 protein, and cluster of differentiation (CD) 56; negative for cytokeratin AE1/AE3, desmin, HMB‐45, CD34, c‐kit, and paired box protein 8 (PAX8); and had a MIB‐1 index of 1%–2% (Figure 2C‐F). GIST was excluded because the tumor cells were negative for c‐kit and CD34, and mild atypia of the tumor with no mitotic activity or necrosis excluded leiomyosarcoma. Renal cell carcinoma with spindle cell change was excluded because the tumor cells were negative for PAX8. Finally, because the tumor cells were negative for HMB‐45 but positive for Melan‐A, we diagnosed fat‐poor leiomyomatous AML. The patient was alive without signs of disease at 12 months postoperatively.

## 3 DISCUSSION

Leiomyomatous AML is composed almost exclusively of smooth muscle cells and a few blood vessels.^2^ AML, whose fat component cannot be confirmed by CT, must be differentiated from renal cell carcinoma. In renal cell carcinoma, the margins are often rounded, whereas in AML, the angular interface, i.e., icecream cone pattern, is useful for differentiation.^7^ However, in the present case, the margin was round and these findings were not observed. Microscopically, lipocytes should be searched for because such renal tumors must be distinguished between leiomyomatous AML and smooth muscle tumors such as leiomyoma, leiomyosarcoma, GIST, or renal cell carcinoma with spindle cell change. HMB‐45 immunostaining of the smooth muscle can help confirm the diagnosis of leiomyomatous AML especially when lipocytes are absent.^2^ Although Bonsib^8^ reported HMB‐45‐positive leiomyoma arising from the renal capsule, this is now considered to be leiomyomatous AML.^1^ Usefulness of HMB‐45 immunostaining in the differential diagnosis of renal tumors composed predominantly of smooth muscle cells was stressed.^1^

Almost all previously reported leiomyomas arising from the renal capsule are now considered to be fat‐poor leiomyomatous AML^1^, ^8^ because nowadays, HMB‐45 has been widely used to diagnose renal smooth muscle tumors, and many of them were positive for HMB‐45 and eventually diagnosed as AML.^1^, ^8^ However, fat‐poor leiomyomatous AML arising from the RP is rare, with only one case reported in detail in English to our knowledge.^6^ Munjal et al.^6^ reported that fat‐poor leiomyomatous AML arising from RP showed spindle cells positive for HMB‐45 that were arranged in whorls in blood vessels and no adipocytes and was similar to leiomyoma, leiomyosarcoma, or GIST. Tumor cells in our patient were positive for both smooth muscle markers and Melan‐A, leading to the diagnosis.

Our patient's tumor cells were negative for c‐kit and CD34, which are frequently expressed in GIST, which excluded metastasis of GIST with smooth muscle differentiation. They were also negative for PAX8, which is frequently expressed in renal cell carcinoma, thus excluding renal cell carcinoma with spindle cell change. However, the cells were positive for smooth muscle markers caldesmon and SMA but negative for HMB‐45. Therefore, we first considered smooth muscle tumors such as leiomyoma and leiomyosarcoma. Roma et al. reported that HMB‐45 and melanocytic marker Melan‐A were sensitive markers for AML positivity 95% and 85% of the time, respectively, that 5% of AMLs were negative for HMB‐45 and positive for Melan‐A, and that combined staining with HMB‐45 and Melan‐A was positive in 100% of AMLs.^9^ Contrastingly, Patil et al.^5^ reported that renal leiomyoma was negative for HMB‐45 and Melan‐A. Our patient's tumor cells were negative for HMB‐45 but positive for Melan‐A, which excluded leiomyoma and led to the present diagnosis. When clinicians encounter HMB‐45‐negative smooth muscle tumors in renal tumors, staining with Melan‐A should be considered. Combined staining with HMB‐45 and Melan‐A in our patient led to the final diagnosis.

## 4 CONCLUSION

Fat‐poor leiomyomatous AML arising from RP without immunoreactivity for HMB‐45 is quite rare. Combined staining with HMB‐45 and Melan‐A may be useful in the differential diagnosis of renal tumors composed predominantly of smooth muscle cells.

## AUTHOR CONTRIBUTIONS

**Yasuyuki Kobayashi:** Conceptualization; data curation; formal analysis; investigation; methodology; project administration; resources; validation; visualization; writing – original draft; writing – review and editing. **Shigeki Shimizu:** Data curation; formal analysis; investigation; methodology. **Hiroki Arai:** Resources. **Kyotaro Yoshida:** Data curation; formal analysis; investigation; methodology. **Masahito Honda:** Supervision.

## FUNDING INFORMATION

This research did not receive any specific grant from funding agencies in the public, commercial, or not‐for‐profit sectors.

## CONFLICT OF INTEREST

The authors declare no conflicts of interest.

## CONSENT

Written informed consent was obtained from the patient for publication of this report.

## Data Availability

No data are available.
